# Detecting and monitoring NO, SNO and nitrite *in vivo*

**DOI:** 10.4155/fso.15.36

**Published:** 2015-08-01

**Authors:** Landon Bellavia, Daniel B Kim-Shapiro, S Bruce King

**Affiliations:** 1 Department of Physics, Wake Forest University, 1834 Wake Forest Rd, Winston-Salem, NC 27106, USA; 2 Department of Physical Science, The University of Findlay, 1000 N Main St, Findlay, OH 45840, USA; 3 Chemistry Department, Wake Forest University, 1834 Wake Forest Rd, Winston-Salem, NC 27106, USA

**Keywords:** chemiluminescence, electron paramagnetic resonance spectroscopy, spin trap

## Abstract

The detection and quantification of nitric oxide and related reactive nitrogen species *in vivo* is vital to the understanding of the pathology and/or treatment of numerous conditions. To that end, several detection and quantification methods have been developed to study NO, as well as its redox relatives, nitrite and *S-*nitrosothiols. While no single technique can offer a complete picture of the nitrogen cycle in a given system *in vivo*, familiarity with the benefits and limitations of several common tools for NO_x_ determination can assist in the development of new diagnostics and therapeutics.

Nitric oxide (NO) is an important bioactive molecule with numerous roles in physiological systems and potential therapeutic applications, many of which are discussed throughout this issue. Understanding the role of NO, particularly in cases where NO homeostasis may be disrupted or NO regulation is intrinsically tied to pathology, relies on the accurate detection and quantification of this molecule. Many techniques exist for both the direct and indirect quantification of NO and related nitrogen species (including nitrite/nitrate and *S-*nitrosothiols, Table 1). Each method has its advantages under certain experimental conditions and with regard to specific targets, but no single method allows for complete, unrestricted quantification of NO *in vivo* (summarized in Table 2). However, depending on the desired test parameters, many techniques or combinations of techniques can be used to collect data on NO concentrations in physiological systems.

## Electrochemical probes

Real-time, *in vivo* generation of NO can be detected by electrochemical sensors. Such probes utilize a system of two or three microelectrodes (a working electrode, a reference electrode and in some systems, an auxiliary electrode) to oxidize NO to NO^+^, which results in a small redox current (in the range of picoAmps to nanoAmps). By measuring this redox current in the system of interest and comparing it to the redox current between the electrodes when calibrated using NO standards, nearly real-time (requiring 30–60 s to equilibrate) NO concentrations can be monitored *in vivo*.

Working electrodes at a potential of 0.8–0.9 V relative to a silver/silver chloride reference electrode oxidize NO to NO^+^. Early electrochemical probes used working electrodes that were either gold-plated or made of pure platinum, though more recently, an alloy of platinum (90%) and iridium (10%) has been suggested due to its improved durability over pure Pt and the electrocatalytic properties of Ir in redox reactions [1]. Alternatively, carbon fiber working electrodes have been shown to have sensitivity comparable to gold-plated electrodes, and have the distinct advantage of being far smaller (˜5 µm diameter) than other electrodes (i.e., ˜130 µm diameter for the Pt-Ir electrode) [2].

Care must be taken with working electrodes to prevent interaction with anions such as nitrite, nitrate, ascorbate or urates; pretreating the electrode with Nafion repels anions. Moreover, oxidation of nonnegative small molecules, such as dopamine and 5-HT, can also result in overestimation of NO levels; this can be partially compensated for by pretreating electrodes in *o*-phenylenediamine to reduce interaction with cations [3].

Unfortunately, the extreme variability between different electrodes casts doubt on the utility of such devices for NO quantification. Different working electrodes can vary by as much as six orders of magnitude when attempting to quantify NO *in vivo*, despite extensive calibration against standards. Recent work suggests that this is due in part to electrode size, with larger electrodes underestimating NO due to rapid NO consumption from an unstirred layer; this work suggests that smaller electrodes are preferable not only for decreased invasiveness, but also for increased accuracy [4].

## Electron paramagnetic resonance

Electron paramagnetic resonance (EPR) spectroscopy is a technique that can, in theory, detect the paramagnetic species in a sample (i.e., species that have one or more unpaired electrons, including free radicals such as NO). However, in practice, several factors may limit the applicability of EPR to any given system. NO is difficult or impossible to detect directly via EPR in most biological systems due to its broad, indistinct EPR signal in aqueous solution. However, it is possible to take advantage of the unique chemical properties of NO in order to create a reaction product, adduct or intermediate with a resolvable EPR spectrum.

Conventional diamagnetic spin trapping agents (e.g., nitrone-based traps), which are widely used to detect transient free radicals, are often not effective traps for NO. However, nitronyl nitroxides (NNOs), most notably carboxy-PTIO (c-PTIO), have been shown to be extremely effective at scavenging and trapping NO, resulting in a chemical shift that allows real-time EPR observation of the scavenging of NO. The efficacy of this assay is strongly dependent on the use of a proper amount of NNO for the experimental conditions. Moreover, both NNO reactants and products are easily reduced in many biological systems to EPR silent species, limiting their utility in quantification of NO production over time. NNOs can also directly interfere with NO generating pathways, or provide new reactive pathways for the generation of NO. These issues, coupled with the fact that NNOs (specifically c-PTIO) have recently been shown to react with other radicals, such as NO_2_, have led to a decrease in the use of NNOs to quantify NO generation *in vivo* via EPR, except under specific conditions [5].

Other families of spin traps exist for the purpose of isolating NO, many of which utilize iron to bind NO and create an adduct that can be detected by EPR. The most common family of synthetic iron-based spin traps are iron-dithiocarbomate-based traps, including diethyldithiocarbamate [6] and *N*-methyl-d-glucamine dithiocarbamate (MGD) [5]. These spin traps have distinct EPR spectra at room temperature, allowing for real-time observation of the trapping of NO, and can be used in conjunction with isotope substitution to identify sources of endogenous NO. However, they oxidize easily under aerobic conditions to create ferric complexes and reactive oxygen species, and may themselves generate NO from HNO, nitrite or *S-*nitrosothiols.

Among the many biological targets for reaction with NO, hemoglobin (Hb) and myoglobin (Mb) are some of the most relevant, and can also be used for the detection and quantification of NO. NO reacts or coordinates with ferrous heme depending on the presence or absence of oxygen. If the ferrous heme is oxygenated (forming oxyhemoglobin, oxyHb), NO reacts to form nitrate and ferric methemoglobin (metHb). MetHb at concentrations aboveapproximately 0.5 µM is easily resolved and quantified by EPR, even in a background with high concentrations of other Hb species, though the NO is consumed in its formation. Because oxyHb reacts very quickly with NO (with a rate constant between 5 and 8 × 10^7^ M^-1^ s^-1^), quantification of metHb formed from oxyHb during a process known to generate NO is often a reliable means of quantifying NO production [7]. If NO is present with a ferrous heme that is not coordinated with a ligand (deoxyhemoglobin, Hb), the NO will coordinate with the Hb to form ferrous nitrosyl hemoglobin (HbNO). HbNO is relatively stable (with a dissociation rate on the order of 10^-3^–10^-4^ s^-1^), and has a distinct EPR signal that can be used to quantify hemoglobin-bound NO at concentrations of approximately 0.5 µM and above [8]. Additionally, because the NO is detected directly, isotope substitution can be used to trace the source of NO. Use of heme proteins in conjunction with EPR is limited by the fact that heme EPR spectra must be collected at cryogenic temperatures, and therefore measurements cannot be taken in real time or directly *in vivo*. In addition, EPR can only detect these reaction products at concentrations of a few hundred nanomolar or more.

## Fluorescence

Beyond detection and quantification of NO in biological systems, direct imaging of NO distribution within systems promises even further insight into the role of this molecule *in vivo*. While several techniques have been developed for the fluorescent detection of NO, many of them have relatively poor detection thresholds and low specificity. Thus, three techniques are most commonly used for the direct imaging of NO *in vivo* [9].

The first successful fluorescent imaging of NO *in vivo* was carried out with diamino-aromatic fluorescent compounds (DAFCs). DAFCs (including DAF, DAF-2, DAR, DAQ, DAF-FM-DA and others) react quantitatively with NO under aerobic physiological conditions to create fluorescent products with absorption and emission spectra in the visible or near-IR range, allowing for detection and imaging of NO concentrations above 5 nM. DAFCs do not react with nitrite and nitrate, allowing for NO imaging amid a background of nitrogen oxides at much higher concentrations than NO [9], nor do they react with peroxide, superoxide or peroxynitrite [10]. However each member of this family has unique advantages and drawbacks, such as pH sensitivity and specificity. The DAFC class of NO detectors is universally limited by the fact that they require oxygen in order to react with NO, limiting utility in hypoxic conditions, as well as by their propensity to react with oxidized NO products (notably N_2_O_3_) and other nitrogen-containing radicals and reducing agents [9].

To overcome many limitations of the DAFCs, a new family of copper (II) based fluorescent probes with sensitivity similar to DAFCs (5 nM) was developed [11] and extensively explored (reviewed in [9]). These copper-based fluorescein derivatives react directly with NO, regardless of local oxygen concentration, and are specific to NO, ignoring other reactive nitrogen species, reactive oxygen species and ascorbate. Cell membranes are generally permeable to these copper-fluorescein molecules, allowing for intercellular and intracellular imaging of NO concentrations. However, they are limited by a suboptimal emission wavelength, as well as potential cytotoxicity and instability *in vivo*. Thus, use of these fluorescent probes has been largely limited to cell cultures, with benzimidazole derivatives having been used in frozen tissue samples.

DAFCs and copper-fluoresceins react with NO irreversibly, and may lead to the buildup of a high level of background fluorescence. To create a reversible NO detector, a fluorescent protein with the NO-specific heme domain of soluble guanylate cyclase was created. While both NO-specific and reversible, the threshold of detection for this molecule was only 10 µM. A related protein system with a much better detection limit (0.1 nM) has been developed for use with FRET imaging, but this requires genetic encoding of the detector into the target organism. Future work in this area may lead to a more ubiquitous fluorescent NO detector, but current sGC- and FRET-related detectors, while they have a few unique advantages, are generally less useful than current DAFCs and copper-fluorescein fluorescent probes [9].

### Nitrite

Nitric oxide generally has a relatively short half-life *in vivo*, ranging anywhere from tens of seconds in some tissues to milliseconds or microseconds in the presence of heme-containing proteins such as hemoglobin. With the correspondingly low steady-state concentration of NO, real-time quantification of NO is difficult and at often times, the concentration of the nitrite and nitrate metabolites of NO will serve just as well as (or better than) the concentrations of NO itself [12]. Moreover, recent advances in NO chemistry have revealed that nitrite constitutes a significant reserve of bioavailable nitric oxide. This nitrite can be reduced to NO under hypoxic conditions by a variety of physiological processes, including acidic reduction and the reaction of nitrite with xanthine oxidoreductase, thiol-containing enzymes and deoxygenated heme proteins such as hemoglobin, myoglobin and neuroglobin. The ability of nitrite to rapidly form NO during hypoxic stress makes it a critical hypoxic buffer responsible for triggering vasodilation as oxygen levels drop [13]. Due to the intrinsic link between nitrite and NO, accurate quantification of nitrite is important to understand systems of hypoxic vasodilation, hypoxic mitochondrial respiration and ischemia-reperfusion and to the development of nitrite-based treatments for conditions characterized by ischemia and hemodynamic dysregulation. To this end, several techniques have been developed or adopted for the quantification of nitrite in biological systems (summarized in Table 3).

## Griess reaction

For over a century, the Griess reaction and its derivatives have been used to quantify nitrite in a variety of situations. However, application of the Griess reaction for *in vivo* quantification of nitrite presents unique challenges. For instance, the typical Griess assay has a detection limit of 1–2 µM, a full order of magnitude above basal nitrite levels in many biological fluids such as plasma. Moreover, the reaction cannot be readily utilized in whole blood due to interference from blood constituents such as hemoglobin and plasma proteins. Thus, various techniques have been developed to improve sensitivity and facilitate blood, plasma and serum sample analysis using techniques based on the Griess reaction, each with advantages and disadvantages. The presence of even trace contaminants, anticoagulants and proteins can all reduce the accuracy of Griess-based assays [14,15].

Numerous sample preparation and storage methods have been used in the analysis of nitrite and nitrate concentrations by the Griess reaction, and some of the procedures used are not clear. Thus, even seemingly similar protocols can differ by an order of magnitude or more in their reported concentrations. Out of the commercially available storage monovettes, serum has been shown to have the lowest levels of nitrite contamination. EDTA and citrate, and to a lesser extent heparin, have been found to have significant levels of nitrite contamination, often greater than the nitrite concentration in the sample itself. Beyond selection of storage media, samples must be properly preserved and prepared for analysis by techniques based on Griess reactions. Nitrite can oxidize forming nitrate, a process that can be inhibited by alkali. Alkali is often combined with ZnSO_4_ to both prevent oxidation of nitrite and precipitate proteins from the solution that could potentially interfere with nitrite detection via nitrosation of cysteine or nitration/nitrosation of carbon or nitrogen under the acidic conditions necessary to conduct the Griess reaction [14]. Dilution of plasma samples in ethanol has also been shown to reduce the effect of plasma proteins on the modified Griess reagents [15]. Due to the importance of deproteination to the success of the Griess assay, additional ultrafiltration or microdialysis techniques may be employed to purify samples, and many deproteination systems are built in to automated Griess assays that also involve other methods, such as chromatography [14].

## Chromatography

Although the Griess reaction is the most widely cited technique for the quantification of nitrite, it is by no means the only method available, nor does it exclude the concurrent use of other techniques to improve the quality of data collection, detection limits and range of conditions under which data can be gathered. Numerous chromatography-based assays exist for the detection of nitrite (and often nitrate), frequently employing pre- or postcolumn treatments (sometimes based on the Griess reaction) to quantify these anions without interference from contaminants in the initial solution [12].

HPLC techniques can be used to quantify nitrite in conjunction with the Griess reaction described above or other methods of nitrite detection, such as the DAN fluorochrome (2,3-diaminonaphthalene) described elsewhere [16]. HPLC systems using either anion-exchange or polystyrene polymer columns have improved on quantification methods relying solely on the Griess reaction, improving sensitivity up to 500-fold [14]. However, the results from such procedures are harder to standardize and can vary between instruments, requiring that great care be taken to validate each use.

Specialized systems designed and marketed for the detection of nitrogen oxides (typically nitrite and nitrate, collectively referred to as NO_x_) employ both HPLC and spectrophotometric analysis of the diazo product of the Griess reaction. These specialized systems have the advantage of highly reproducible results that can be obtained with less time-consuming sample preparation procedures. However, as with any commercial assay system, care must be taken to validate the assay in each new situation and to ensure consistent quality of results [12].

In addition to HPLC techniques, assays based on GC-MS have also been developed, with pentafluorobenzyl bromide (PFB-Br) used for the simultaneous derivatization and quantization of nitrite [12,17]. Recent work has used these techniques to quantify nitrite in whole blood rather than plasma or serum [17].

## Chemiluminescence

Ozone-based chemiluminescence is a technique for the detection of NO gas that has been used with great success in a variety of applications. It is particularly useful in the determination of *in vivo* NO metabolites such as nitrite and nitrate, and can be used in situations where traditional Griess reaction assays and chromatography fail. This typically requires a specialized chemiluminescent detector, and such detectors designed for the quantification of NO are commercially available and well validated [18].

While the potential applications of chemiluminescence are numerous, it is a particularly valuable tool for the determination of nitrite and nitrate (as well as *S-*nitrosothiols, as discussed later in this article). Notably, chemiluminescence does not require deproteination prior to analysis, and can be conducted in the presence of hemoglobin and other contaminants, although oxidizing hemoglobin before analysis (e.g., through the use of ferricyanide) has been shown to be useful in the prevention of oxidation of nitrite to nitrate [19].

Chemiluminescent determination of nitrite or nitrate requires the reduction of the relevant NO_x_ to NO within an anaerobic reaction vessel. Numerous reducing agents can be used, and different agents will reduce different NO_x_ species; for example, tri-iodide (I_3_^-^) acidified with acetic acid can be used to reduce the majority of relevant NO_x_ species and is preferred for analysis of nitrite concentration (in addition to using potassium iodide alone), whereas copper(I)/cysteine is often used for the determination of *S-*nitrosothiols (discussed later in this issue).

Chemiluminescence is highly sensitive, with a detection threshold on the order of 1 pmol of total nitrite or 1 nM of nitrite in solution, depending on the quantity of sample used. However, it also has limitations and potential pitfalls. Foaming (especially when using copper(I)/cysteine as a reductant) can cause an overflow from the reaction vessel when biological samples (particularly those with high protein levels) are injected into the reaction chamber. There is also a great deal of variability between instruments, and even on the same instrument between uses; thus, care must be taken to calibrate the instruments against a set of known standards before each use [18].

### *S-*nitrosothiols

In addition to existing freely in solution or being oxidized to another species entirely, NO occurs *in vivo* as a protein modification factor. NO in the presence of oxygen can form *S-*nitrosothiols (R-S-N=O) (RSNOs). These RSNOs may transport NO activity, and also likely affect cellular NO signaling pathways. Thus, determination of the *in vivo* concentration of these RSNOs is important for understanding NO transport and signaling within the body. Quantification of small-molecule RSNOs at relatively high concentration *in vitro* can often be accomplished using UV/Vis spectroscopy, but the relatively low concentrations of these compounds *in vivo*, coupled with the fact that *S-*nitrosothiols of interest are often associated with large macromolecules, render spectroscopic techniques untenable. Thus, the most commonly used techniques for quantification of RSNOs generated *in vivo* rely on detection of liberated NO or the labeling of thiols vacated following the liberation of NO (summarized in Table 4).

## Chemiluminescence

As described earlier, chemiluminescence can be used to detect the liberation of NO due to the reduction of nitrogen oxides. Chemiluminescent detection has been used to quantify NO release from the reduction of *S-*nitrosated proteins formed *in vivo*. Three reducing systems currently employed to facilitate this chemiluminescent detection assay are summarized here, and have been previously reviewed in greater detail [20].

In the tri-iodide (I_3_^-^) method, potassium iodide (KI) and iodine (I_2_) create tri-iodide in the presence of acetic acid. This serves as the reducing agent, simultaneously liberating NO from RSNOs, nitrosamines, iron–nitrosyl complexes and nitrite. Because of the less-specific nature of this assay, and the frequent contamination of materials by nitrite, care must be taken to maintain accurate controls [18].

Another system uses copper chloride, cysteine and carbon monoxide (the so-called 3C method). In this technique, Cu(I) reduces the nitrosothiol, while cysteine both reduces Cu(II) and forms CSNO. CO gas flow prevents any heme present from capturing NO before it can be detected. This method has the significant advantage of being nitrite silent, and thus ignores many otherwise-troublesome sources of contamination [21]. The 3C method is also convenient in that it requires no pretreatment of samples, though the difficulties caused by the use of CO may offset this advantage. CO, beyond the obvious health risks, can cause instrumental complications, such as overheating in some models of chemiluminescent detectors (notably in the hopcolite filter of the Sievers Nitric Oxide Analyzer, NOA 280i, which can be compensated for by placing ice packs around the filter). Protein-containing samples can also cause excessive foaming, requiring frequent replacement of the purge vessel solution and often making this procedure more time consuming than a comparable tri-iodide assay [20].

To overcome some of the difficulties with the 3C assay, a modified copper/cysteine procedure has been developed, frequently referred to as the modified 2C method. This uses pretreatment with K_3_Fe(CN)_6_, NEM and DTPA. K_3_Fe(CN)_6_ (ferricyanide) oxidizes hemoglobin to the non-NO capturing metHb state, while NEM blocks free thiols from becoming *S-*nitrosated during the treatment, and the metal chelator DTPA protects SNO bonds from free metals. The modified 2C method eliminates the complications of CO flow during the experiment, while preserving many of the 3C method's advantages (particularly nitrite independence). However, this method requires pretreatment of samples and additional care when dealing with some hemoglobin-based samples [20].

Each of these methods has advantages and disadvantages, but all three share certain key features. Perhaps most notably, while chemiluminescence can allow for accurate quantification of NO liberated from *S-*nitrosothiol reduction, it does not provide any insight into the specific location of that NO. For this, other techniques must be used, and can often be used in tandem with chemiluminescence for improved results.

## Biotin switch technique

The most widely used method for the indirect detection and labeling of *S-*nitrosothiols in proteins is the biotin switch technique (BST). This technique involves three basic steps. First, the free cysteine thiols must be blocked (e.g., by NEM, *MMTS* or IAM); next, RSNOs are reduced (typically by ascorbate); finally, free thiols are tagged with a biotin label or other compound, such as a fluorescent marker, that facilitates separation of labeled proteins by techniques like western blotting or biotin pull-down. However, there are several concerns surrounding this technique, notably, the possibility that some sites of *S-*nitrosation are destroyed during the procedure, and the potential lack of specificity and completeness of ascorbate in reducing RSNO, with indications that ascorbate may not reduce RSNOs at all sites, and may also reduce sulfenic acids and some disulfides. Despite these concerns, the BST has been widely used to identify SNO nitrosation sites, with many recent variations offering improved sensitivity and specificity. These various modified versions of the BST are collectively a topic of some length, and have been widely reviewed [22–24].

Examples of notable recent advances in BST-related assays include the development of a microarray-based assay, which uses an antibiotin antibody to facilitate high-throughput analysis with great sensitivity. Although this technique does not cover the entire proteome, and can return a relatively few false positives, it can provide rapid insight into target proteins of *S-*nitrosation by various NO releasing compounds, including those that may be developed for therapeutic applications. Another method, termed the d-Switch technique, has recently been developed for analysis of cell lysates. This technique uses NEM to block and tag all free thiols, followed by a procedure similar to the BST to reduce *S-*nitrosothiols and label them with isotope labeled d5-NEM. This technique allows quantification of both total cysteine and *S-*nitrosated cysteine by LC–MS. Such systems allow for identification of *S-*nitrosated proteins directly from cell lysate. While this is not an *in vivo* assay, the ability to identify proteins as candidates for *S-*nitrosation can provide valuable insights into physiological systems, and contribute to models of biological activity [22–24].

## Fluorescence

RSNOs can also be detected by fluorescent methods. In general, reactive species such as RSNOs may be targets of less-specific fluorescent probes [10]. While fluorescent detection methods specific to RSNO have yet to be developed into techniques that are compatible with *in vivo* analysis or imaging, promising initial steps in this direction have been taken. For instance, a coumarin-based phosphine was recently developed that fluoresces upon reaction of the phosphine with RSNO [25]. This molecule promisingly shows no reactivity with disulfides, although its general specificity for RSNOs has not yet been documented. It may also lack the necessary level of sensitivity, having only provided quantitative measurements of RSNO in the low mM range *in vitro*. However, the potential of such molecules, including the family of triarylphosphines [23], to serve as RSNO probes, coupled with the addition of fluorescent indicators such as this coumarin- phosphine, suggests that direct fluorescent detection of RSNOs *in vivo* may be attainable [24]. A recent report describes a mass spectrometric method for detecting small molecule *S-*nitrosothiols based on this phosphine chemistry [26].

## Conclusion & future perspective

The accurate detection and monitoring of NO, nitrite and *S-*nitrosothiols *in vivo* present numerous challenges, which depend largely on the conditions of study and the inherent difficulty in tracking transient species with short lifespans and relatively low physiological concentrations. While by no means an exhaustive list, many of the most useful and most widely used techniques, as well as some promising candidates for future investigation, have been summarized here. The interested researcher is encouraged to seek more details on any methods of interest in the articles referenced here. In the future improvements in fluorescence-based or mass-spectrometry-based techniques may improve in vivo detection. For now, there are many available techniques all of which can provide useful information provided proper controls are incorporated.

**Table 1.** **Nitrogen species and their oxidation states.**

**Compound, structure**	**Oxidation state**
Nitrate, NO_3_^-^	+5
Nitrogen dioxide, NO_2_	+4
Nitrite, NO_2_^-^	+3
Nitric oxide, NO	+2
Nitroxyl, HNO	+1
Nitrogen, N_2_	0
Hydroxylamine, NH_2_OH	-1
Ammonia, NH_3_	-3

**Table 2.** **Benefits and limitations of select techniques for determination of nitric oxide.**

**NO techniques**	**Benefits**	**Limitations**
Electrochemical probes	*In situ* data	Up to 10^6^ difference between techniques
	Nearly real time	Electrode fragility and fouling
		Electrode pretreatment requirements
EPR: NNOs	Highly effective NO trap	Nonspecificity
	Distinct spectral shift between NO-bound and unbound states	Reduced to EPR silent species under some biological conditions
EPR: iron-dithiocarbomate spin traps	Room temperature	Prone to oxidation
	Real-time observation of NO trapping	Best under anaerobic conditions
	Isotope substitution compatible	May generate (and artificially detect) NO from HNO, nitrite, *S-*nitrosothiols
EPR: metHb	Quantifiable, with relatively low (500 nM) limit of detection	Aerobic conditions required
	Detectable amid high concentrations of hemes and free radicals	Cryogenic temperatures (˜5K)
		NO irreversibly consumed in reaction
		Does not directly detect NO
EPR: HbNO	NO trapped	Partially oxygenated conditions only
	Isotope substitution compatible	May detect nitrite as well
Fluorescence: DAFCs	NO (or N_2_O_3_) imaging in cells	Aerobic conditions required
	Sensitivity: 5 nM detection limit	Reacts with oxidized NO metabolites, especially N_2_O_3_
		Generates N_2_O_3_ intermediate
Fluorescence: copper fluoresceins	Direct NO imaging in cells	Suboptimal emission wavelength
	No N_2_O_3_ intermediate	Cytotoxicity
	Sensitivity: 5 nM detection limit	Potential instability in biological systems
Fluorescence: FRET	Reversible	Detection limit of 10 µM for nongenetically modified cells
	NO detection limit of 0.1 nM in genetically modified cells	

DAFC: Diamino-aromatic fluorescent compound; EPR: Electron paramagnetic resonance; FRET: Fluorescence resonance enery transfer; metHb: Methemoglobin; HbNO: Nitrosyl hemoglobin.

**Table 3.** **Benefits and limitations of select techniques for determination of nitrite.**

**Nitrite techniques**	**Benefits**	**Limitations**
Griess	Quantifiable reaction with nitrite	High limit of detection (1–2 µM) in assays using only Griess methods
	Provides a basis for modified assays	Susceptible to contamination
Chromatography	Can be used in conjunction with Griess technique, fluorochromes and others	More difficult to standardize
	Improves sensitivity up to 500×	Variation between instruments
	Eliminates some pretreatment needs	
Chemiluminescence	Does not require deproteination	Specificity (potentially detects other NO_x_ species)
	Very high sensitivity (˜1 nM)	Frequent calibration required
		Variation between instruments

**Table 4.** **Benefits and limitations of selected techniques for determination of *S-*nitrosothiols.**

***S-*nitrosothiol techniques**	**Benefits**	**Limitations**
Chemiluminescence: tri-iodide	Quantifies NO release	Specificity
	Less foaming and overheating compared with other reducing agents	Must employ sulfanilamide to eliminate nitrite contamination
		Does not reveal location of *S-*nitrosation
Chemiluminescence: 3C	Quantifies NO release	Carbon monoxide risks
	Nitrite silent	Instrument overheating
		Excess proteins cause foaming
		Does not reveal location of *S-*nitrosation
Chemiluminescence: modified 2C	Quantifies NO release	Requires pretreatment of sample
	Nitrite silent	Excess proteins cause foaming
		Does not reveal location of *S-*nitrosation
Biotin switch techniques	Labels site of *S-*nitrosation	Indirect detection method
	Adaptable to advances in the state-of-the-art, for example, microarray-based assays and d-switch technique	Some *S-*nitrosation sites may be destroyed in process
		Ascorbate may lack some specificity
		May detect other modified thiols
Fluorescence	Potential to develop RSNO-specific fluorescent tags	Not yet validated for specificity to RSNO or for work at low concentrations
	Potential to combine with mass spectrometry	Not yet applicable *in vivo*

RSNO: Nitrosothiol, where R denotes an organic group.

**Executive summary**

There are many ways to investigate nitric oxide, nitrite and *S-*nitrosothiols *in vivo*, but none of them are universally applicable in all situations.
NO can be determined by direct *in vivo* use of electrochemical probes (though these are subject to many limitations), electron paramagnetic resonance spectroscopy and fluorescence imaging.
Nitrite can be determined by use of the Griess reaction and its derivatives, chromatography and chemiluminescence.
*S-*nitrosothiols can also be quantified by chemiluminescence; determination of their location on proteins requires tagging the sites of *S-*nitrosation, such as by the Biotin switch technique or a derivative thereof.
